# Are figure legends sufficient? Evaluating the contribution of associated text to biomedical figure comprehension

**DOI:** 10.1186/1747-5333-4-1

**Published:** 2009-01-06

**Authors:** Hong Yu, Shashank Agarwal, Mark Johnston, Aaron Cohen

**Affiliations:** 1 Department of Health Sciences, University of Wisconsin-Milwaukee, P.O. Box 413, Milwaukee, WI 53201-0413, USA; 2 Department of Computer Science, College of Engineering, University of Wisconsin-Milwaukee, P.O. Box 413, Milwaukee, WI 53201-0413, USA; 3 Medical Informatics, College of Engineering, University of Wisconsin-Milwaukee, P.O. Box 413, Milwaukee, WI 53201-0413, USA; 4 Department of Occupation Therapy, College of Health Sciences, Oregon Health & Science University, 3181 S.W. Sam Jackson Park Road, Portland, Oregon, USA 97239-3098, USA; 5 Department of Medical Informatics and Clinical Epidemiology, School of Medicine, Oregon Health & Science University, 3181 S.W. Sam Jackson Park Road, Portland, Oregon, 97239-3098, USA

## Abstract

**Background:**

Biomedical scientists need to access figures to validate research facts and to formulate or to test novel research hypotheses. However, figures are difficult to comprehend without associated text (e.g., figure legend and other reference text). We are developing automated systems to extract the relevant explanatory information along with figures extracted from full text articles. Such systems could be very useful in improving figure retrieval and in reducing the workload of biomedical scientists, who otherwise have to retrieve and read the entire full-text journal article to determine which figures are relevant to their research. As a crucial step, we studied the importance of associated text in biomedical figure comprehension.

**Methods:**

Twenty subjects evaluated three figure-text combinations: figure+legend, figure+legend+title+abstract, and figure+full-text. Using a Likert scale, each subject scored each figure+text according to the extent to which the subject thought he/she understood the meaning of the figure and the confidence in providing the assigned score. Additionally, each subject entered a free text summary for each figure-text. We identified missing information using indicator words present within the text summaries. Both the Likert scores and the missing information were statistically analyzed for differences among the figure-text types. We also evaluated the quality of text summaries with the text-summarization evaluation method the ROUGE score.

**Results:**

Our results showed statistically significant differences in figure comprehension when varying levels of text were provided. When the full-text article is not available, presenting just the figure+legend left biomedical researchers lacking 39–68% of the information about a figure as compared to having complete figure comprehension; adding the title and abstract improved the situation, but still left biomedical researchers missing 30% of the information. When the full-text article is available, figure comprehension increased to 86–97%; this indicates that researchers felt that only 3–14% of the necessary information for full figure comprehension was missing when full text was available to them. Clearly there is information in the abstract and in the full text that biomedical scientists deem important for understanding the figures that appear in full-text biomedical articles.

**Conclusion:**

We conclude that the texts that appear in full-text biomedical articles are useful for understanding the meaning of a figure, and an effective figure-mining system needs to unlock the information beyond figure legend. Our work provides important guidance to the figure mining systems that extract information only from figure and figure legend.

## Background

The biomedical literature is a rich resource of biomedical knowledge. The importance of developing valid information retrieval systems for biomedical scientists has motivated the development of domain-specific information systems and annotated databases worldwide. However, most information retrieval systems target only textual information and fail to provide access to other important data contained in journal articles such as image data, including figures. More than any other type of information, figures usually represent the evidence of discovery in the biomedical literature [1]. Full-text biomedical journal articles nearly always incorporate figures. For example, we found an average of five figures per biomedical article in the journal *Proceedings of the National Academy of Sciences *(PNAS) [1]. Biomedical scientists need to access figures to validate research facts and to formulate or test novel research hypotheses. Evaluation has shown that textual statements reported in the literature are frequently noisy (i.e., contain "false facts") [2]. Therefore, access to the experimental evidence in the form of figures is an important aspect of scientific communication and peer review.

Unfortunately, this wealth of information remains highly underutilized and inaccessible without automatic systems to mine these figures. A recent review article [3] describes emerging research interest in mining figures. Specifically, the subcellular location image finder (SLIF) system [4,5] extracts information from fluorescence microscopy images and aligns image panels to their corresponding sub-legend. Rafkind et al. [6] integrated text features in figure legend with image features for figure classification. Shatkay et al. [7] integrated image features with text to enhance document classification. BioText [8,9] indexes figure legends and returns figure+legend in response to a text query.

The work described above, however, explored only figure legends, and not other types of associated text. As described in [1,10,11], the figure legend is not the only type of text that describes figure content. The other types of associated text include the title, the abstract, and other kinds of text that appear in full-text articles. Although numerous studies have shown the importance of figure legend for literature-mining [1,6,8-14], the importance of other types of associated text has not yet been evaluated. The question we raise is that, are other associated texts important, and if so, to what extend, for understanding the meaning of figures? Is it necessary for a figure mining system to ignore other associated texts?

The questions we raised are of important value. Because biomedical full-text articles are highly structured, titles and figure legend are typically easy to detect. In contrast, detecting the associated texts that appear in the abstract and in the full-text body is a much harder task, and requires sophisticated natural language processing approaches (NLP) we have recently developed [1]. A recent study showed that seven of the eight biologists who used the BioText figure-legend retrieval system said that the BioText legend search was useful [8], note that the study didn't explore other associated texts. Many journals (e.g., Nature) have requested authors to make the figure legend comprehensive. If figure legend is sufficient for figure-mining tasks, then the need for sophisticated NLP techniques for automatically linking figures to other associated texts may be limited.

Therefore before designing automated systems to specifically augment figure understanding with full text, it is essential to determine the quantitative difference in figure understanding to be gained by providing access to the relevant portions of the full text article as compared to the simpler alternatives of legend, title, and abstract.

In this study, we identify what types of text are necessary for biomedical scientists to understand the meaning of a figure that appears in a full-text biomedical article. We have previously observed that associated texts in abstracts are better than other types of associated text, including figure legend, for summarizing figure content [10]. We hypothesize that in addition to legend, the texts that appear in full-text biomedical articles are useful for understanding the meaning of a figure. We have designed a randomized study to systematically evaluate whether texts other than figure legend are important aids to biomedical researchers in figure comprehension.

## Methods

We designed a randomized trial protocol to test whether figure comprehension would increase with incremental levels of associated text. The evaluation protocol was approved by the University of Wisconsin-Milwaukee Institutional Review Board (IRB).

### Test conditions

Three types of figure-text combinations were compared: (1) figure+legend, (2) figure+legend+title+abstract, and (3) figure+full-text.

### Subject

We recruited a total of 25 subjects by email and online advertisements (e.g., Nature Jobs). All subjects were bench biomedical scientists who were either PhD candidates or post-docs in the biomedical domain. The subjects' specialties include biological science, cell biology, ecology, genetics, pathology, physiology, plant, molecular biology, and structural biology. We consulted five subjects on methods for quantitatively measuring figure comprehension. The rest of the 20 subjects participated in the evaluation. The subjects self-reported that they frequently search literature resources for their research. Subjects were paid for their participation (~$10/hr).

### Figure Selection

We selected evaluating figures from a representative biomedical literature collection, the TREC 2006 Genomics Track text collection, which was derived from 49 biomedical journals and includes a total of 162,259 full-text biomedical articles [15].

To select figures for evaluation, we randomized the order of the articles in the Genomics Track text collection, and picked the first figure of the correct type from the first article for each of the five defined figure types (i.e., gel image, graph, image-of-a-thing, model, and mixed type) [6]. No more than one figure was selected from a given full-text article. We continued this process in a round-robin fashion until we had selected a total of 25 figures from 25 full-text articles. These 25 full-text articles belonged to 9 journals. Our strategy for selecting articles and figures ensured that the articles and figures represented a randomized collection of biomedical literature and figures in genomics.

### Measures

Previous work has shown that figure comprehension can be measured quantitatively [16]. By consulting with five biomedical scientists, we developed two measures for figure comprehension. First, we measured figure comprehension on a Likert-like scale (SCORE): we asked subjects to give a score between 1 and 10 (poorest and best, respectively) to indicate the extent she/he understood the figure's meaning (MEANING) when different figure-text combinations were provided, and also to give a score between 1 and 10 (poorest and best, respectively) to indicate their confidence in providing the assigned score (CONFIDENCE). There were no time constraints during the evaluation process.

Second, we measured figure comprehension by evaluating the text summaries (TEXT-SUMMARY) for each figure-text combination. We asked each subject to write a text summary to describe the content of the figure, and we then quantitatively analyzed the text summaries. Previous work has shown that biomedical text can be classified into different rhetorical units including *Background*, *Methods*, *Results*, and *Conclusions and Indications *[17,18]. We hypothesized that a text summary of a figure can also be structured by rhetorical units.

To test this hypothesis, we consulted five subjects about what types of information were important for representing figure content. We provided each subject with all the rhetorical units defined in other studies (e.g., [17,18]), and asked the subject to select the rhetorical units that can be used to structure the meaning of a figure. We also asked each subject to freely enter any other types of rhetorical units. All five subjects selected the following four rhetorical units: purpose of the study, methods, results, and conclusions and indications, and commented that each unit can be described with text. These four rhetorical units were then used for evaluating figure comprehension.

We implemented a web-based user interface to allow the 20 subjects to view a figure+text combination and to score it by MEANING and by CONFIDENCE. Additionally, subjects were told to enter free-text summaries on the basis of the four rhetorical units (purpose, methods, results, conclusions and indications). In addition to the rhetorical units, for each figure and figure-text combination we provided a text box for each subject to enter "other important criteria for understanding the figure content." An example of the free text provided by the subjects is shown in Table 1. Again, we did not impose any time constraints to subjects who completed the evaluation study.

**Table 1:** Sample data for rhetorical unit evaluation.

Figure + Associated text	Subject-Generated Text Structured by Rhetorical Units				
	
	Purpose	Methods	Results	Conclusions	Other Criteria
Fig+L	Effect of pH...	Enzyme kinetics	Hard to say, need detailed fitting data	Don't know	Full text

Fig+L+T+A	The goal of...	X-ray crystallography...	Hydrogen bonds...	RNA binding of protein...	Methods are still lacking

F+Legend+T+A	To show that...	A is just a chart of...	Affinity of TF...	A model for the formation...	How was the final model arrived at (part c)?

Space limitations prevent us from including all the text descriptions for the four rhetorical units. Legend (L), title (T), and abstract (A). The original data are available at http://www.bioex.us.com/evaluation_data/JudgeEvaluation.xml.

### Procedures

Each subject of the 20 total was presented with five figures, one of each of the five figure types. Each figure was randomly selected from the five figures that belonged to a specific figure type. The order of the figure presentation was randomized. For each figure, the subject was shown first the figure+legend, then the figure+legend+title+abstract, and finally the figure plus the full-text article. At each level of figure text, the subject was asked to assign scores by MEANING and CONFIDENCE, and was requested to enter a summary text for each rhetorical unit and for other important criteria for comprehending figures. There was no time limit for a subject to complete the evaluation for any figure-text type.

### SCORE Analysis

We analyzed the SCORE results using McNemar's test on paired samples, where the subject and figure were the same and only the amount of associated text differed. As is commonly done with Likert data, the SCORE samples were thresholded into two dichotomous groups. Since we are concerned with scientists having a high level of figure comprehension, scores less than 8 were considered to represent poor understanding of the figure on the part of the subject. A score of 8 or greater represented good understanding of the figure on the part of the subject. By thresholding the data into two groups, distinctions between strong understanding and a lesser level of understanding could be statistically analyzed in a simple and robust manner.

### MISSING INFORMATION Analysis

We applied a straightforward approach to quantitatively evaluate the quality of text summaries of figures. We counted, with each figure-text, whether a text summary indicated that information was missing. A text summary was counted as containing missing information if the text incorporated cue text such as "don't know," "don't understand," "no clue," "lacking," "missing," "hard to say," "requires more," etc. Since each text summary is structured into the four rhetorical units, we counted whether information was missing in each of the rhetorical units separately. For example, Table 1 shows that for the figure+legend, the results, and the conclusions and indications were missing. For the two data samples shown for the figure+legend+title+abstract combination, the methods are missing in the first, and both the results, and the conclusions and indications are missing for the second.

We generated a coding guideline based on the cue text. A rhetorical unit was judged as complete (not missing any required information) if none of the cue text was present. We then asked two biomedical scientists (PhD in biology) who were not among the 20 subjects to serve as judges. Following the coding guidelines, the two judges independently identified whether there was information missing from the text summary of each of the subject/figure/text/rhetorical-unit combinations. The judges provided a dichotomous rating: missing or complete (non-missing). We blinded the judges to the figure-text combination information, the figure type information, and the information about which subjects wrote the summaries. The data were randomized for order presentation to each judge. We measured the inter-rater agreement between the two judges by the measures of overall agreement and Cohen's kappa [19]. As with the SCORE data, the relationships between test conditions and dichotomized comprehension scores were tested using McNemar's test on paired samples, where the subject and figure were the same and only the amount of associated text differed.

### ROUGE Score Analysis

Another approach to evaluating the quality of text summaries is to apply the methods developed for summarization evaluation tasks [20]. Ideally, summaries should be assessed either by human judgments on their quality and utility [21] or by a task-based setting to determine their usefulness as part of an information browsing and access interface (extrinsic evaluation) [22,23]. However, such evaluations are time-consuming, expensive, and require a considerable amount of time and careful planning.

Automatic summarization methods have been developed to reduce the bottleneck of human intervention. *Recall-oriented Understudy for Gisting Evaluation *(ROUGE) is the most frequently used automated summary evaluation package, and has been used in Document Understanding Conference (DUC) [24]. A summary is evaluated by comparing it with a human-generated gold standard based on the computation of n-gram overlap between the summary and the gold standard. An n-gram is a subsequence of n items (words) from a given text span. An n-gram of size 1 is a "unigram" (1-gram); one of size 2 is a "bigram" (2-gram), etc.

A high-quality gold standard is crucial to the ROUGE evaluation. In our study, biomedical scientists generated all the text summaries. However, for each figure, the text summaries were generated with different figure-text combinations. Since the full text provides the subjects with the most complete descriptions of figures, it is reasonable to assume that the full-text-figure combination is the closest (among the three) to the gold standard. We therefore applied the text summaries generated by use of the figure+full-text as the gold standard, and obtained the ROUGE scores by comparing them to other text summaries generated by using other figure-text combinations. We evaluated the text summaries by rhetorical unit. In addition, we aggregated the four rhetorical-unit text summaries of a figure to form one full-text summary and evaluated that full-text summary. Each text summary was evaluated against the gold standard for the same figure. Since, for each figure, four subjects evaluated the same figure+full-text combination, we applied one subject's summary as a gold standard to evaluate the three other subjects' judgments. By doing so, we can compare the ROUGE scores among all three figure-text types.

The ROUGE package [24] uses recall-based metrics based on n-gram matching between candidate summaries and reference summaries. The package has numerous parameters, including stemming (a process for reducing inflected words to their root form), stop-word removal (to filter out common words such as "a", "the", etc.), and the choice of n-gram. ROUGE-n (n is a number) is n-gram recall; ROUGE-L is based on the longest common subsequence (the common subsequence with maximum length); and ROUGE-W is a weighted longest common subsequence that takes into account distances when applying the longest common subsequence. Different settings reportedly work better for different summarization tasks [24] and, therefore, different parameters need to be tested for new tasks. In our evaluation, we computed different settings (ROUGE-1, ROUGE-2, ROUGE-L, and ROUGE-W [weight = 1.2]) and explored stemming and stop-word removal.

## Results and Discussion

We report two different approaches to evaluate figure comprehension. First we asked 20 subjects to enter scores based on their self-evaluation of their understanding. Secondly, we asked the same subjects to enter text to summarize figure content. We then evaluated the text summaries of those figures for estimating figure comprehension using two methods. We identified whether the text summary incorporated any cue text that indicated information in the text summary was missing, and applied automatic summarization evaluation techniques to evaluate the quality of text summaries.

In all, 16 subjects completed both the SCORE and MISSING INFORMATION tasks; this corresponds to the total of 240 subject/figure/figure-text combinations (16 × 5 × 3). Two subjects completed one and two subject/figure/figure-text combinations, respectively. Two subjects dropped out because of their time availability. The total number of subject/figure/figure-text combinations on which we collected data was 243. We found that the average (± SD) time spent for a subject to complete the task was 25.7 ± 70.4 min for figure+caption, 33.6 ± 147.7 min for figure+caption+title+abstract, and 20.0 ± 26.9 min for figure+full-text. Since all evaluation was completed online and the evaluators were not instructed to complete the evaluation task within a block of time, it is not surprising that there were wide confidence intervals. We did not find statistically significant differences in time spent among various figure-text combinations. Note that because of our sequential evaluation design, i.e., we show each evaluator first figure+caption, then figure+caption+title+abstract, and finally the full-text article, we can not exclude that there are statistical differences among different figure-text combinations.

Table 2 shows the average score for SCORE and for CONFIDENCE. Table 3 shows the McNemar testing of SCORE and CONFIDENCE. The results showed that there were significant differences between all pairs of text conditions for SCORE. Figure+legend+title+abstract was significantly different from figure+legend at p ≤ 0.001. Figure+full-text was significantly different from figure+legend at p ≤ 0.0001. Figure+legend+title+abstract was significantly different from figure+full-text at p = 0.027. The CONFIDENCE analysis using McNemar testing showed a significant difference between Figure+full-text and figure+legend at p = 0.006, but not between the other two text pairings.

**Table 2:** Score of comprehension and Confidence for each Text Type

Text Type	Score of comprehension	Confidence
Fig+L	5.82 ± 2.90	7.63 ± 2.28

Fig+L+T+A	7.41 ± 2.19	8.17 ± 1.70

Full text	8.17 ± 1.97	8.46 ± 1.81

**Table 3:** The P-value of McNemar test of SCORE and CONFIDENCE

Text Type 1	Text Type 2	Binary SCORE	Binary CONFIDENCE
Fig+L	Fig+L+T+A	0.0006	0.2120

Fig+L	Full text	p ≤ 0.0001	0.0055

Fig+L+T+A	Full text	0.0271	0.0995

Table 2 shows that both comprehension and confidence of comprehension increase when more associated text are available. Using the image+caption category as a baseline, the score for figure comprehension increased 27.3% when title and abstract were added; when full-text was available, the score increased 40.4%. As shown in Table 3, the McNemar testing on SCORE concluded that there were significant differences between all pairs of figure-text types when we asked subjects to give a score to indicate to what extent she/he understood the figure. This is clear evidence that the amount of associated text is important to a scientist's subjective understanding of a biomedical figure. This was true across all increments of text. The increase from legend to legend+title+abstract helped the subjects somewhat, but the full text was necessary to achieve the highest levels of understanding consistently.

Table 4 shows the average number of words in the summaries written by the subjects for the purpose, methods, results, and conclusions in different figure-text combinations. When other associated text are provided in addition to figure legend, our data show that the number of words significantly increase (p ≤ 0.0001, paired t-test) in three rhetorical units (except for Methods); between legend and full-text, the number of words increases significantly (p ≤ 0.0001, paired t-test) in all four rhetorical units. Between legend+title+abstract and full-text, the number of words increases (p ≤ 0.0002, paired t-test) in Methods.

**Table 4:** Average number of words and standard deviation in annotator summaries

	Purpose	Methods	Results	Conclusions
Fig+L	11 ± 8	8 ± 11	18 ± 18	8 ± 7

Fig+L+T+A	18 ± 11	10 ± 13	45 ± 40	21 ± 15

Full text	18 ± 11	18 ± 13	44 ± 35	26 ± 19

We found statistical differences in number of words among the four rhetorical units, and did not find any statistically significant word-number differences between any pair of the five figure types (data not shown). During the evaluation subjects frequently commented that the full text, as well as other related figures, was important for understanding the content of a figure. Clearly, subjects in general needed more words to describe the figure when they had more associated text available to them. This implies that the subjects learned new information that they thought was important to the summary as they read more text.

Table 5 shows the inter-rater agreement between the two judges for the MISSING INFORMATION analysis task. The two judges had a kappa agreement of greater than 0.75 for identifying information missing from the figure+legend+title+abstract in the Methods and Conclusions, and information missing from the figure+legend in the Purpose and Methods. The two judges had a kappa agreement of 0.40–0.75 for the rest of text-figure-rhetorical-unit combinations. Kappa values of 0.40–0.75 represent fair to good agreement beyond chance, and values greater than 0.75 represent excellent agreement [19].

**Table 5:** Inter-rater pairwise and kappa agreement

	Purpose Missing	Method Missing	Result Missing	Conclusion Missing
Fig+L	89.87% (0.79)	93.67% (0.87)	79.75% (0.59)	86.08% (0.66)

Fig+L+T+A	96.15% (0.71)	93.59% (0.83)	87.18% (0.65)	91.03% (0.78)

Full text	98.68% (0.66)	93.42% (0.58)	88.16% (0.40)	88.16% (0.54)

Our results show a good inter-rater agreement (kappa ≥ 0.4) between two judges for missing information comprehension; this demonstrates the effectiveness of applying cue text for identifying missing information from the text summaries. We found that the inconsistency was mainly introduced by differences in interpreting the information in the "other important criteria" field. The inconsistency can be contributed by the ambiguity introduced by the subject who entered the text, the inconsistency in interpreting the text, and challenges in separating related information. For example, when a subject questioned about the type of cell used, as in the example of "*what type of cells are they using? (S. cerevisiae isn't mentioned*)", one could interpret that information is missing in Method. Accordingly, when the methods are missing, the purpose, results and conclusions may also be indicated as unclear as well, because purpose motivates methods, methods inform the interpretation of results, and results inform the conclusions.

We manually examined the text summaries to determine what caused the disagreement between the judges. We found that two judges agreed entirely when missing information was identified by cue text within a rhetorical unit. All disagreements were introduced by the differences in interpreting the text in "other important criteria" entered by subjects. For example, one judge considered that information was missing in the Purpose, while the other judge interpreted as missing in Method when the text in "other important criteria" was "*What type of cells are they using? (S. cerevisiae isn't mentioned*)." When the "other important criteria" was "background of this protein," one judge considered that information was missing in Purpose, while the other judge did not.

Tables 6 and 7 show the p-values of McNemar's test for significant differences between figure-text types on dependent rhetorical unit variables for judges 1 and 2, respectively. We found that for both judges, the differences in comprehension as the amount of associated text increased were highly significant for all four rhetorical units when comparing figure+legend with either figure+legend+title+abstract or figure+full-text (p < 0.05). Comparing figure+legend+title+abstract to figure+full-text revealed significant differences for missing methods (judge 1, p = 0.004, and judge 2, p = 0.0099), and for missing conclusions (judge 2, p = 0.0246). The results further support that both comprehension and confidence of comprehension increase when more associated texts are available.

**Table 6:** P-value of McNemar's test for differences between figure – text types (judge 1)

Text Type 1	Text Type 2	Purpose Missing	Method Missing	Result Missing	Conclusion Missing
Fig+L	Fig+L+T+A	0.0000	0.0154	0.0004	0.0000

Fig+L	Full Text	0.0000	0.0000	0.0000	0.0000

Fig+L+T+A	Full Text	0.4868	0.0043	0.0851	0.1910

**Table 7:** P-value of McNemar's test score for differences between figure – text types (judge 2)

Text Type 1	Text Type 2	Purpose Missing	Method Missing	Result Missing	Conclusion Missing
Fig+L	Fig+L+T+A	0.0000	0.0205	0.0000	0.0000

Fig+L	Full text	0.0000	0.0000	0.0000	0.0000

Fig+L+T+A	Full text	0.2300	0.0099	0.0533	0.0246

The figure comprehension rates by the INFORMATION MISSING results are shown in Table 8. We found that the differences in comprehension as the amount of associated text increased were highly significant for all four rhetorical units (p ≤ 0.0001, chi-square test for trend). Post hoc pair-wise Fisher's exact analysis showed that comprehension associated with full text was significantly greater than figure+legend for all four rhetorical units (p ≤ 0.001) and better than figure+legend+title+abstract for Methods, Results, and Conclusions (p ≤ 0.05 with Bonferroni correction). When the full-text article is not available, presenting just the figure+legend left biomedical researchers lacking 39–68% of the information about a figure as compared to having complete figure comprehension; adding the title and abstract improved the situation, but still left biomedical researchers missing 30% of the information. When the full-text article is available, figure comprehension increased to 86–97%; this indicates that researchers felt that only 3–14% of the necessary information for full figure comprehension was missing when full text was available to them. Clearly there is information in the abstract and in the full text that biomedical scientists deem important for understanding the figures that appear in full-text biomedical articles.

**Table 8:** Comprehension rates (non-missing rhetorical units) associated with figure – text types for four rhetorical units.

	Fig+L	Fig+L+T+A	Full Text
Purpose	0.57	0.92	0.97

Methods	0.61	0.76	0.91

Results	0.49	0.76	0.89

Conclusions	0.32	0.73	0.86

Table 9 lists F-scores of the ROUGE-L measurement. We evaluated the text summaries by rhetorical unit. We also aggregated the four units to form a full summary and evaluated the full summaries. Each text summary was evaluated against figure+full-text judgments for the same figure. For summaries generated with the figure+full-text combination, one subject's summary was measured against other subjects' judgments. The score of a figure-text combination is the average of all summaries or rhetorical units of that category. The table shows that the quality of summaries or rhetorical units largely depends on the figure-text combinations. For the figure+legend combination, all four rhetorical units and full summaries received the lowest scores. By adding the title+abstract, average F-scores increased, and the full-text significantly improved the scores. This is consistent with the other evaluations of the differences among the different figure-text combinations. We also computed ROUGE-1, ROUGE-2, and ROUGE-W (data not shown), and the trend for these did not differ from ROUGE-L.

**Table 9:** ROUGE-L F-scores for different figure – text types.

	Fig+L	Fig+L+T+A	Full text
Purpose	0.46	0.60	0.61

Methods	0.38	0.48	0.59

Results	0.28	0.35	0.58

Conclusions	0.22	0.28	0.56

Full summaries	0.32	0.40	0.58

The ROUGE analysis shows that for the figure+legend combination, all four rhetorical units and full summaries received the lowest ROUGE scores. By adding the title+abstract, the average F-scores increased, and the full text significantly improved the scores. For example, for the full summaries, the F-score for the full summary increased 25%, from 0.32 in figure+legend to 0.40 in figure+legend+title+abstract, and further increased 45%, to 0.58 in figure+full-text. The differences are statistically significant. The results support that the quality of text summaries increases when the amount of associated text increases.

On the other hand, the overall ROUGE scores in our study are relatively low compared to the DUC ROUGE evaluation results [16]. This is not surprising, because ROUGE measures whether a particular summary has the same words (or n-grams) as a reference summary. ROUGE is mainly used for extractive summarization evaluation where summaries include only sentences selected from the original texts. However, in our experiments, summaries are not extractive. Instead, they are generated by subjects without constraints on word choice. It is very common that summaries of almost identical meanings have very different words. This results in low ROUGE scores. As our purpose is to measure the difference between different combinations rather than summaries of one particular combination, ROUGE is still an effective measure in our evaluation, although it might not be the best one. An alternative is to apply the pyramid evaluation [17]. The pyramid method differs from ROUGE primarily in assigning weights to summary content units, not bags of words. A content unit represents a minimum semantic unit in a text summary. To carry out the pyramid method, we will need to recruit additional biomedical domain experts to manually analyze the text summaries by content unit. Obviously this is much more labor intensive than the method used in our study and remains for future work.

Analyzing by content unit is an informative approach and will continue to be part of our future work when researching text-summary-based figure comprehension. When we manually examined the text summaries generated by biomedical experts, we found that both the depth and breath of information in a text summary increase as more associated text is provided. An example is shown in the following which is a list of text summaries (shown within quotation markers) generated by one subject with different figure-text types on Figure 2 in the article [25]:

**Figure+legend**: *Purpose: *"Compare growth rates between wild type and ybr159 mutant cells." *Methods: *"Measure the growth rate with photometer." *Results: *"Growth rate of the mutant cells is lower." *Conclusions: *"The maximum growth in wild type cells occurs earlier."

**Fig+L+T+A**: *Purpose: *"Examine effect of Ybr159w disruption in S. cerevisiae mutant cells." *Methods: *"Disruption of Ybr159w gene, compare growth between wild type and mutant cells via optical method." *Results: *"Mutants are slow growing and display high temperature sensitivity." *Conclusions: *"Disruption of YBR159w is not lethal since there is some growth in mutant cells."

**Full text**: *Purpose: *"Identify a gene required for the reconstitution of heterologous elongase activity. Examine the requirement of YBR159w for yeast viability." *Methods: *"they used a loss-of-heterologous-function screen to genetically identify a component of the microsomal fatty acid elongase." *Results: *"cells are able to grow at 30°C in rich medium (in the absence of fatty acid supplements) but at a slower rate than wild type." *Conclusions: *"initial slow growth may be related to some form of adaptive response to the loss of this microsomal elongase component. After this adaptation the mutant spore colonies formed are viable in the absence of fatty acid supplement, although growing at a much slower rate than wild type cells."

As shown in the text summaries above, it is clear that both the quantity and quality of text summaries increase as more associated text was provided. However, such increase was implicit in our study, based on the lack of evidence of misunderstanding; the subject did not enter any symbolic cue text to indicate any missing information in text summaries. An advantage of our binary coding scheme was its simplicity and consistency. However, a multipoint rating or more complex and detailed coding guidelines may be necessary to detect more subtle differences in missing information and improve the analysis. Ultimately, a semantic interpretation analyzing text summaries by content unit may be the best approach to measure the level of figure comprehension. If we can accurately measure the implicit missing information, we speculate that the differences in figure comprehension will be even higher among different figure-text types.

One limitation in our evaluation design is that the associated text was given to each subject incrementally. The advantage of such an evaluation design is that we can support the statistical validity with much fewer examples than needed for a randomized evaluation using paired comparisons. On the other hand, the design may introduce a bias: the longer a subject is exposed to the figure, the better the subject understood the figure. We think that such a bias was minimized in our study because we did not pose any time constraint on each subject for his/her evaluation at each level of text. In fact, our data show that there was no statistical difference in the time spent among different types of associated text. Furthermore, many summaries, such as the one shown above, clearly demonstrate that the difference in missing information was caused by the content of associated text available to the subject, and not by the exposure time given to a figure.

In summary, all the evaluation approaches used in this study strongly indicate that there are statistically significant differences in biomedical researchers' figure comprehension when they are given three different levels of text – the legend, legend+title+abstract, and full-text. Clearly, the figure+legend is insufficient, the figure+legend+title+abstract is somewhat better, but having access to the full text article is necessary to really understand the full meaning of a figure.

## Conclusion

We conclude that associated text other than the figure legend is very important for biomedical scientists' understanding of the meaning of a figure in a full-text biomedical article. Systems that ignore the information in the full-text article, and that only present to users abstract and figure legend could risk losing 30% of information in figure comprehension. We predict that automated systems that extract the relevant explanatory information or a summary from the full text along with extracted figures could be very useful in reducing the workload of biomedical scientists who would otherwise have to retrieve and read the associated full-text journal article to determine which figures are relevant to their research and understand the biomedical evidence presented in those figures.

## Authors' contributions

HY and AC designed the experiments. MJ participated in the experiment design. SA carried out the experiments with subjects. HY wrote the paper. AC reviewed and edited the paper.
